# Serum Levels of the Cytokine TWEAK Are Associated with Metabolic Status in Patients with Prostate Cancer and Modulate Cancer Cell Lipid Metabolism In Vitro

**DOI:** 10.3390/cancers13184688

**Published:** 2021-09-18

**Authors:** Antonio Altuna-Coy, Xavier Ruiz-Plazas, Marta Alves-Santiago, José Segarra-Tomás, Matilde R. Chacón

**Affiliations:** 1 Disease Biomarkers and Molecular Mechanisms Group, IISPV, Joan XXIII University Hospital, Universitat Rovira i Virgili, 43007 Tarragona, Spain; antonio.altuna@iispv.cat (A.A.-C.); xruiz.hj23.ics@gencat.cat (X.R.-P.); malves.hj23.ics@gencat.cat (M.A.-S.); 2 Urology Unit, Joan XXIII University Hospital, 43005 Tarragona, Spain

**Keywords:** TWEAK, prostate cancer, lipid metabolism, Fn14

## Abstract

**Simple Summary:**

TWEAK is an inflammatory cytokine related to prostate cancer (PCa) progression that exerts its effects by engaging its cognate receptor Fn14. A soluble form of TWEAK (sTWEAK) has been detected in the PCa microenvironment. Altered levels of circulating sTWEAK are associated with aberrant glucose metabolism. We show that reduced serum levels of sTWEAK are associated with the metabolic status in patients with PCa and that the treatment of PC-3 cells with sTWEAK enhances the expression of genes related to lipid, but not to glucose, metabolism. sTWEAK also increases the lipid uptake and lipid accumulation in PC-3 cells. We corroborated that the observed effects were due to TWEAK/Fn14 engagement by silencing Fn14 expression, which attenuated the aberrant gene and protein expression. Additionally, we observed that the phosphorylation of ERK1/2 and AKT (ser473) were required for TWEAK/Fn14 actions. Thus, the contribution of the sTWEAK/Fn14 axis on PCa metabolism supports its potential as a therapeutic target for PCa.

**Abstract:**

Soluble TWEAK (sTWEAK) has been proposed as a prognostic biomarker of prostate cancer (PCa). We found that reduced serum levels of sTWEAK, together with higher levels of prostate-specific antigen and a higher HOMA-IR index, are independent predictors of PCa. We also showed that sTWEAK stimulus failed to alter the expression of glucose transporter genes (*SLC2A4* and *SLC2A1*), but significantly reduced the expression of glucose metabolism-related genes (*PFK*, *HK1* and *PDK4*) in PCa cells. The sTWEAK stimulation of PC-3 cells significantly increased the expression of the genes related to lipogenesis (*ACACA* and *FASN*), lipolysis (*CPT1A* and *PNPLA2*), lipid transport (*FABP4* and *CD36*) and lipid regulation (*SREBP-1* and *PPARG*) and increased the lipid uptake. Silencing the TWEAK receptor (Fn14) in PC-3 cells confirmed the observed lipid metabolic effects, as shown by the downregulation of *ACACA*, *FASN*, *CPT1A*, *PNPLA2*, *FABP4*, *CD36*, *SREBP*-*1* and *PPARG* expression, which was paralleled by a reduction of *FASN*, *CPT1A* and *FABP4* protein expression. Specific-signaling inhibitor assays show that ERK1/2 and AKT (ser473) phosphorylation can regulate lipid metabolism-related genes in PCa cells, pointing to the AKT locus as a possible target for PCa. Overall, our data support sTWEAK/Fn14 axis as a potential therapeutic target for PCa.

## 1. Introduction

Prostate cancer (PCa) is the second-most commonly diagnosed cancer in men worldwide [1]. A central challenge in the management of PCa is discriminating between the indolent and aggressive disease, and the early detection of PCa is important to guide treatment strategies. In this context, the prostate-specific antigen (PSA) test, as well as the tumor, node, metastasis (TNM) classification and the Gleason score in prostate biopsies, are routinely used for predicting the prognosis and informing treatment decisions [2]. The current treatments for PCa include radical prostatectomy, performed as a curative therapy, and androgen deprivation treatment, which is typically achieved using androgen receptor antagonists.

Solid tumors employ aerobic glycolysis to generate energy (the so-called Warburg effect). To support transformation, cancer cells can rewire their metabolism to sustain the production of energy, and, in this sense, fatty acid metabolism has recently received particular attention. Cancer cells can fuel their fatty acid metabolism through the activation of pathways such as the PI3K-AKT-mTORC1 signaling axis [3]. Fatty acids can be obtained from de novo lipogenesis and from exogenous uptake, which are facilitated by transporters such as CD36 (cluster of differentiation 36) and FABP (fatty acid-binding protein). Fatty acids can be stored as lipid droplets and used for acetyl-CoA production through β-oxidation. De novo lipogenesis relies on the enzymatic activities of ACLY (ATP citrate synthase), ACACA (acetyl-CoA carboxylase alpha) and FASN (fatty acid synthase), which generate diverse lipid species [3,4].

Aberrancies in cellular metabolism in PCa are recognized hallmarks of malignant transformation [5]. Indeed, the large amount of citrate that would normally be secreted functions as an intermediate in the citric acid cycle and as a substrate for de novo fatty acid synthesis. In this regard, the dysregulation of lipid metabolism involving the upregulation of lipogenic enzymes and of enzymes that function to oxidize fatty acids as an energy source is a common finding in PCa [6]. Furthermore, a recent systematic review and meta-analysis found that patients with PCa had higher fasting serum insulin and higher homeostasis model assessment-estimated insulin resistance (HOMA-IR) levels, especially those over 65 years of age [7]. Insulin is a known growth factor for PCa cells [8]; however, its role in PCa etiology remains unclear.

A strong association is known to exist between chronic inflammation and several types of cancer, and inflammatory cytokines have been considered as potential mediators of prostate carcinogenesis [9]. Yet, the modulatory actions of the inflammatory factors on energy metabolism in prostate epithelial cells have been poorly investigated.

The tumor necrosis factor-like weak inducer of apoptosis (TWEAK) is a cytokine expressed mainly by monocytes and macrophages [10]. TWEAK can exist as a full-length, membrane-anchored form (mTWEAK) on the cell surface [10] or as a biologically active soluble form (sTWEAK) generated by the proteolytic processing of mTWEAK, which is detectable in the extracellular milieu and biofluids [11]. Both forms functionally engage the fibroblast growth factor-inducible 14 (Fn14) receptor to activate downstream signaling pathways [12,13]. Early studies on the TWEAK/Fn14 axis in PCa identified Fn14 as a candidate for enhanced PCa growth in xenografted mice under high-fat feeding [14] and with a probable role in invasion, migration and proliferation in PCa cell models [15]. Similarly, the overexpression of Fn14 contributes to multiple malignant cellular phenotypes associated with cancer progression in androgen-independent PCa cell lines, in part controlled by the tissue remodeling enzyme matrix metalloproteinase 9 (MMP-9) [15]. The finding of a potential relationship between the Fn14 expression and patient outcome in patients undergoing radical prostatectomy for localized PCa [15] points to Fn14 as a novel prognostic biomarker candidate in PCa. In an effort to identify new PCa biomarkers in liquid biopsies, we recently established that semen levels of sTWEAK were lower in high-risk patients than in low-risk peers and that this reduction was accompanied by a trend for an increase in the Fn14 mRNA expression levels in seminal cell sediment [16]. More recently, we found that sTWEAK semen levels and TWEAK-induced oncogenic shuttle microRNAs could be integrated into an improved PCa prognostic panel based only on information obtained from semen [17].

sTWEAK serum levels are significantly and independently associated with an increased risk of type 2 diabetes and metabolic syndrome [18], collectively highlighting a possible role for sTWEAK in the pathogenesis of metabolic diseases. This is supported by the finding that sTWEAK can modulate in vitro human hepatocyte lipid accumulation [19].

Given the established relationship between sTWEAK and metabolic-related pathologies, we sought to study whether there was a possible link between circulating sTWEAK levels and the metabolic status of patients with PCa. To this end, we investigated sTWEAK serum levels in a well-characterized cohort of patients with PCa in relation to glucose and lipid metabolic profiles. Additionally, to better understand the impact of inflammation on metabolic dysregulation in PCa, we used cell models to investigate whether this cytokine, previously reported to be present in the prostate microenvironment, could alter local glucose and/or lipid metabolism in PCa.

## 2. Materials and Methods

### 2.1. Study Population

This was a cross-sectional, case–control, retrospective study of 76 patients with PCa and 159 controls. Control patients with PSA levels below average for their age (<1.3 ng/mL PSA with ages 60–69 years; <1.7 ng/mL PSA with ages 70–79 years) [20] were recruited from outpatients in the hospital Joan XXIII, Tarragona, Spain. Patients with PCa, recruited from the same hospital, had undergone radical prostatectomy, which was supported by the European guidelines and was a treatment option in our Urology Department. The surgeries were performed between 2015 and 2020. The grades, groups and stages of the tumors were determined according to the 2014 International Society of Urological Pathology (ISUP) Gleason Grading (GG) and TNM classification. The patients were stratified into two groups, according to the ISUP GG: low-risk (ISUP Groups I and II) and high-risk (ISUP Groups III, IV and V). This study was performed according to the provisions of the Declaration of Helsinki and was approved by our local ethics committee and adhered to the current legal regulations (Biomedical Research Law 14/2007, Royal Decree of Biobanks 1716/2011 and Organic Law 15/1999 of September 13 Protection of Personal Data). All methods were approved and performed in accordance with the guidelines and regulations of the Ethical Committee for Clinical Research (CEIM) from the Pere Virgili Research Institute (Ref. CEIM171/2017; CEIM205/2020 approved on 02/10/2017 and 24/09/2020, respectively).

### 2.2. Biochemical Data

All patients underwent anthropometric measurements. Blood was extracted for measurements of plasma lipids, glucose, insulin and sTWEAK after an 8-h fast. The glucose and lipid levels were determined by standard laboratory methods. Serum insulin was measured in duplicate using a monoclonal immunoradiometric assay (Medgenix Diagnostics, Fleunes, Belgium). The serum γ-glutamyltransferase (GGT), uric acid and creatinine levels were determined using enzymatic methods. The HOMA-IR index was calculated using the following formula:

serum glucose (mmol/L) × serum insulin (mU/L)/22.5.(1)

Serum concentrations of sTWEAK were determined in duplicate by the enzyme-linked immunosorbent assay using the human TWEAK/TNFSF12 Kit #DY1090 (R&D Systems Europe, Abingdon, Oxon, UK). The intra- and inter-assay coefficients of variation were 2.5% and 7.0%, respectively, and the sensitivity of the test was 62 pg/mL.

### 2.3. Cell Culture and Treatments

The prostate cancer cell lines PC-3 (androgen-insensitive) and LNCaP (androgen-sensitive) and the histologically normal prostate epithelial RWPE-1 cell line (immortalized with papilloma virus 18) were purchased from Sigma-Aldrich (Barcelona, Spain). PC-3 cells were cultured in Ham’s F-12K (Kaighn’s) Medium (1:1 mixture) with L-glutamate (Invitrogen/Gibco, Fisher Scientific SL, Madrid, Spain). LNCaP cells were cultured in RPMI 1640 medium (Merck KGaA, Darmstadt, Germany) supplemented with 1-mM sodium pyruvate (Gibco). PC-3 and LNCaP cultures were supplemented with 10% fetal bovine serum. RWPE-1 cells were cultured in keratinocyte serum-free medium plus 5 μg/mL bovine pituitary extract. All the cells were supplemented with 1× antibiotic-antimycotic (Gibco) and 5 μg/mL Plasmocin^TM^ (InvivoGen, San Diego, CA, USA). Where indicated, the cells were grown in serum-deprived medium overnight before stimulation for 24 or 48 h with 100 ng/mL human recombinant TWEAK (PeproTech, bioNova cientifica, Barcelona, Spain) and were cultured in a humidified 5% CO_2_ atmosphere at 37 °C.

### 2.4. siRNA Experiments

An siRNA targeting the human Fn14 gene (*TNFRSF12A* siRNA sequence sense 5′-GAGGGAGAAUUUAUUAAUATT-3′ and antisense 5′-UAUUAAUAAAUUCUCCCUC-3′) and an siRNA control (MOCK) were purchased from Thermo Fisher Scientific (Invitrogen, Madrid, Spain). The transfected siRNAs concentration used was 50 nM, as recommended by the manufacturer, and has been tested elsewhere [21]. Opti-MEM and Lipofectamine 3000 were used for the transfection (Thermo Fisher Scientific). Twenty-four hours after the transfection, the cells were stimulated or not with 100 ng/mL human recombinant TWEAK for 48 h and then collected for gene expression and protein analysis.

### 2.5. Inhibitor Treatments

The MEK inhibitor PD184352 (Sigma-Aldrich, St. Louis, MO, USA) was used at a final concentration of 10 μM, which led to successful p-ERK inhibition [22]. The AKT inhibitor MK2206 (Deltaclon S.L., Madrid, Spain) was used at a final concentration of 1 µM [23]. Parthenolide (Sigma-Aldrich) and Amgen16 (Sigma-Aldrich) were used for NF-ĸB canonical and noncanonical inhibition, respectively, at 10 µM [24,25]. PC-3 cells were treated with inhibitors for 1 h before stimulation with 100 ng/mL TWEAK (or vehicle) for 24 h. RNA was then extracted for gene expression analysis.

### 2.6. Determination of Fatty Acid Uptake and Lipid Accumulation

PC-3 cells were seeded in 96-well clear flat-bottom black plates, and treatments with sTWEAK and with siFn14 were performed as described above. To quantify the fatty acid uptake, the growth medium was exchanged with TF2-C12 fatty acid (Sigma-Aldrich), and the cells were incubated at 37 °C for 30 min. The cellular uptake was measured on a Varioskan Lux Reader (Thermo Fisher Scientific) by the measurement of fluorescence intensity (*λ*ex = 485/*λ*em = 515 nm). The lipid content was measured with Nile Red (Sigma-Aldrich). Briefly, the media were removed, cells were washed twice with PBS and lipids were stained with 1.1 mg/mL Nile Red for 15 min at 37 °C. After incubation, the cells were washed with PBS, followed by measurement of the fluorescence intensity (*λ*ex = 488/*λ*em = 590 nm).

### 2.7. Gene Expression Analysis

RNA was extracted, cDNA was synthesized, and real-time quantitative PCR was performed on a 7900HT Fast Real-Time PCR system (Applied Biosystems, Foster City, CA, USA), as described [26]. Gene quantification was performed using the following commercial Taqman assays (Applied Biosystems): *ACACA*, *ACLY*, *FASN*, *CPT1*, *PNPLA2*, *SREBP-1*, *PPARG*, *FABP4*, *CD36*, *SLC2A1*, *SLC2A4*, *HK1*, *PFKM*, *PDK4*, *PDHA1* and *PKM2*. The cycle threshold (CT) value for each sample was normalized to the expression of *PPIA* (peptidylprolyl isomerase A), used as an internal control. SDS software (2.3, Applied Biosystems, Foster City, California, USA) and RQ Manager 1.2 (Applied Biosystems) were used to analyze the results with the comparative CT method (2^−∆∆CT^).

### 2.8. Western Blot Analysis

A total of 25 µg of protein was fractionated on 4–15% gradient SDS-PAGE gels and transferred to nitrocellulose membranes using the standard protocols. The following primary antibodies were used: p-ERK1/2 (#4370), p-p38 (#4511), p-JNK (#4668), p-IKB (#2859), NF-kB2 p100/p52 (#4882), Fn14 (#4403), p-AktS473 (#9271), FASN (#3180S) and CPT-1A (#12252) and were purchased from Cell Signaling Technology (Danvers, MA, USA), FABP4 (SC18661) was purchased from Santa Cruz Biotechnology (Dallas, TX, USA) and anti-ß-actin was purchased from Sigma-Aldrich. The antibodies were used at the dilutions recommended by the manufacturers.

### 2.9. Statistical Analysis

For the clinical, anthropometrical and analytical parameters, all data were tested for normality using the Shapiro–Wilk test. Normally distributed data were reported as the mean and standard deviation (SD). Differences between patient groups were tested with the Mann–Whitney *U* test. Spearman’s correlation coefficients were used to analyze the relationship between normally and non-normally distributed parameters. Multiple logistic regression analyses were employed (stepwise backward selection procedures) to determine whether the sTWEAK levels were associated with the presence of PCa. In vitro experimental results were presented as the mean and standard error of the mean (SEM) of 3 to 4 independent experiments. The differences were tested with the unpaired two-tailed Student’s *t*-test. The statistical software SPSS 21.0 (IBM, Madrid, Spain) and R package (R Core Team (2013). R: A language and environment for statistical computing. R Foundation for Statistical Computing, Vienna, Austria) were used for the analyses. Significance was considered at a *p*-value < 0.05.

## 3. Results

### 3.1. Low Concentrations of sTWEAK Are Associated with PCa

**Figure 1:**
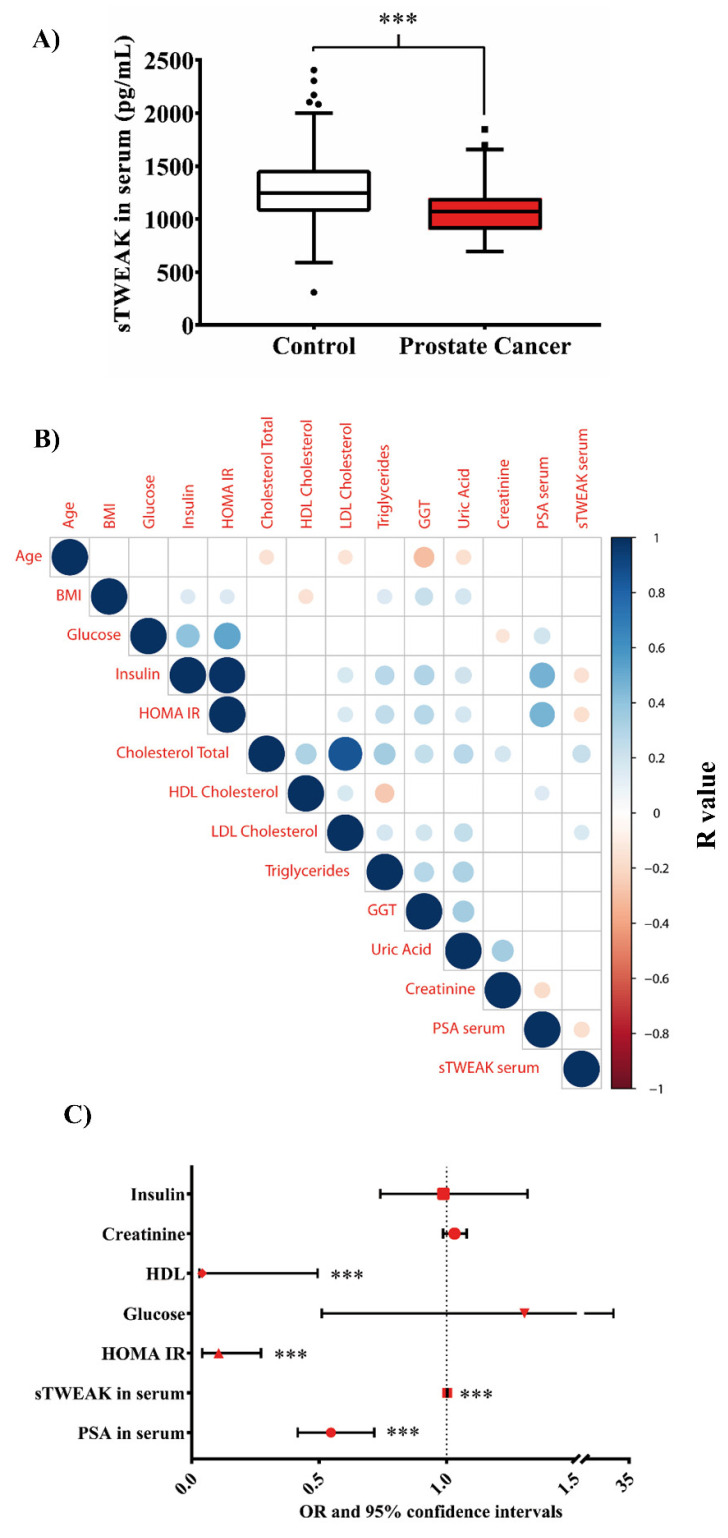
Circulating serum levels of sTWEAK in PCa. (**A**) Box plot (each box shows the median, quartiles and extreme values) representation of the sTWEAK serum levels in the control and PCa cohorts. *** *p* < 0.001. (**B**) Spearman’s correlation matrix. Positive correlations are displayed in graded blue colors and negative correlations in graded red colors. Correlations with *p*-values ≥ 0.05 are considered as insignificant and are left blank. The color intensity and the size of the circle are proportional to the correlation coefficients. On the right side of the correlogram, the color legend shows the correlation coefficients and the corresponding colors. (**C**) Relationship between PCa and clinical, anthropometric and analytical parameters. Odds ratios (OR values in different red shapes for each parameter) and 95% confidence intervals. Dotted horizontal line: OR greater than 1 indicates that variable is more likely to occur in PCa and, an OR less than 1 indicates that variable is less likely to occur in PCa. *** *p* < 0.001. Abbreviations: BMI, body mass index; HOMA-IR, homeostatic model assessment for insulin resistance; HDL, high-density lipoprotein; LDL, low-density lipoprotein; GGT, gamma glutamyltransferase; PSA, prostate-specific antigen; sTWEAK, soluble tumor necrosis factor-like weak inducer of apoptosis.

The baseline characteristics of the 76 patients with PCa and the 159 controls included in the present study are shown in Table 1. Fasting plasma glucose, serum insulin, HOMA-IR, HDL-cholesterol, creatinine and total serum PSA levels were all significantly higher in patients with PCa than in the controls (Table 1), whereas the opposite was seen for the serum sTWEAK levels (Figure 1A). Spearman’s correlation coefficients are described in Figure 1B. The most relevant associations were the negative associations between the sTWEAK and serum PSA (*r* = −0.178, *p* = 0.006), insulin (*r* = −0.156, *p* = 0.017) and HOMA-IR (*r* = −0.164; *p* = 0.012) and the positive associations with the total cholesterol (*r* = 0.234; *p* < 0.001) and LDL cholesterol (*r* = 0.163, *p* = 0.012). Additionally, the serum PSA levels were positively associated with fasting glucose (*r* = 0.194, *p* = 0.003), insulin (*r* = 0.473, *p* < 0.001), HOMA-IR (*r* = 0.465, *p* < 0.001) levels and HDL cholesterol (*r* = 0.149, *p* = 0.022) and negatively associated with serum creatinine (*r* = −0.172, *p* = 0.008).

A multivariate ordered logistic regression analysis was used to evaluate the independent predictors associated with PCa in the whole population. The variables included in the final model were the insulin, creatinine, HDL cholesterol, glucose, HOMA-IR, sTWEAK and PSA levels. The results showed that HOMA-IR (odds ratio (OR) = 0.126, *p* < 0.001), PSA serum levels (OR = 0.539, *p* < 0.001), HDL cholesterol (OR = 0.040 *p* < 0.001) and lower sTWEAK serum levels (OR = 1.002 *p* < 0.001) were independently associated with the presence of PCa (Figure 1C).

### 3.2. sTWEAK Treatment of PCa Cell Lines Alters the Expression of Genes Related to Lipid Metabolism but Not to Glucose Metabolism

**Figure 2:**
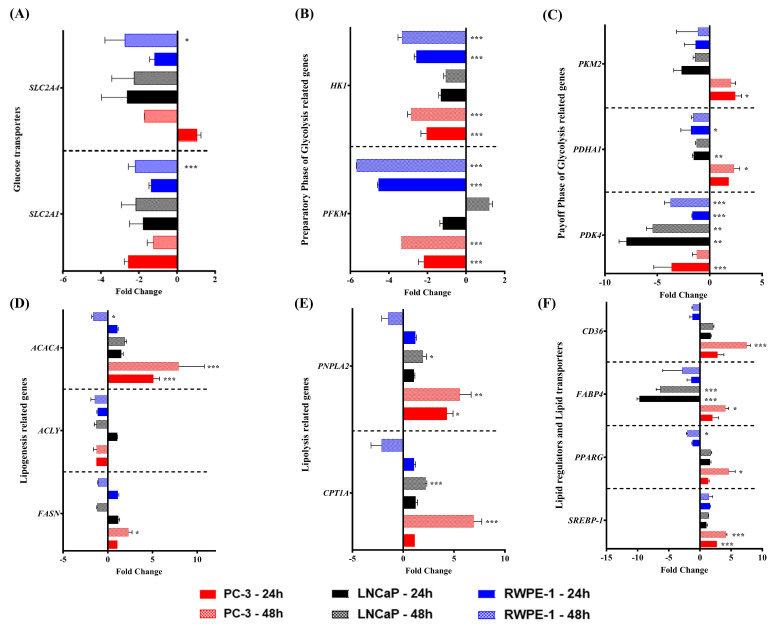
Effect of exogenous sTWEAK on the expression of the genes related to glucose and lipid metabolism. The glucose and lipid metabolism-related genes were evaluated by RT-qPCR after treating PC-3, LNCaP and RWPE-1 cells with 100 ng/mL TWEAK for 24 or 48 h. (**A**) Glucose transporters. (**B**) Preparatory phase of glycolysis. (**C**) Payoff phase of glycolysis. (**D**) Lipogenesis-related genes. (**E**) Lipolysis-related genes. (**F**) Lipid regulators and lipid transporter genes. Results are presented as the mean ± SEM of 4 independent experiments of the fold change expression with respect to the nontreated respective controls. * *p* < 0.05, ** *p* < 0.01 and *** *p* < 0.001. Abbreviations: *SLC2A1* and *SLC2A4*, glucose transporter 1 and 4, respectively; *HK1*, hexokinase 1; *PFKM*, 6-phosphofructo-1-kinase; *PKM2*, pyruvate kinase M2; *PDHA1*, pyruvate dehydrogenase alpha 1; *PDK4*, pyruvate dehydrogenase kinase 4; *ACACA*, acetyl-CoA carboxylase alpha; *ACLY*, ATP-citrate synthase; *FASN*, fatty acid synthase; *PNPLA2*, patatin-like phospholipase domain containing 2; *CPT1A*, carnitine palmitoyltransferase 1A; *CD36*, CD36 molecule; *FABP4*, fatty acid-binding protein 4; *PPARG*, peroxisome proliferator-activated receptor gamma; *SREBP-1*, sterol regulatory element-binding transcription factor 1; PC-3, androgen-independent human advanced adenocarcinoma prostate cancer cell line, LNCaP, androgen-sensitive human prostate adenocarcinoma cell line; RWPE-1 epithelial cell line derived from the peripheral zone of a histologically normal adult human prostate.

To assess whether sTWEAK influences PCa cell metabolism, we examined for possible changes elicited by sTWEAK on the expression of the selected genes associated with glucose metabolism in two PCa cell lines (LNCaP and PC-3 cells) and a control, epithelial cell line (RWPE-1). All cell types expressed the gene for Fn14, which was upregulated under sTWEAK stimulation (Figure S1). The cell cultures were treated with sTWEAK for 24 and 48 h. The results showed that the expression of *SLC2A1* and *SLC2A4* (glucose transporters 1 and 4) decreased significantly in RWPE-1 cells after treatment with sTWEAK (48 h) but not in PC-3 and LNCaP cells (Figure 2A). Gene activity in the “preparatory phase” (energy consuming) of glycolysis was lower in PC-3 and RWPE-1 cells after a 24- and 48-h sTWEAK treatment, as shown by a significant decrease in the expression of hexokinase 1 (*HK1*) and 6-phosphofructo-1-kinase (*PFKM*), whereas no significant changes were observed in LNCaP cells (Figure 2B).

We also examined the “payoff phase” (energy production) of glycolysis, which showed a differential response to sTWEAK in the PCa cell lines studied. Pyruvate kinase M2 (*PKM2*) gene expression was unchanged in LNCaP and control RWPE-1 cells, whereas pyruvate dehydrogenase alpha 1 (*PDHA1*) gene expression was significantly decreased at 24 h. By contrast, the gene expression of *PKM2* and *PDHA1* significantly increased at 24 and 48 h, respectively, in PC-3 cells. Additionally, the expression of pyruvate dehydrogenase kinase 4 (*PDK4*) was significantly lower in LNCaP cells at 24 h and in RWPE-1 cells at 24 and 48 h after TWEAK treatment (Figure 2C).

The downregulation of *PDK4* and concomitant upregulation of *PDHA1* in PC-3 cells is noteworthy, as *PDK4* is an inhibitor of *PDHA1*, which might indicate that the pyruvate generated by PKM2 is converted into acetyl-CoA by the action of PDHA1, thus entering the citric acid cycle and fueling lipogenesis in PC-3 cells.

Next, we investigated whether sTWEAK administration altered the expression of the genes involved in lipid metabolism in PCa cell lines. We found that sTWEAK treatment significantly increased the expression levels of lipogenesis-related genes such as acetyl-CoA carboxylase alpha (*ACACA*) at 24 and 48 h and fatty acid synthase (*FASN*) at 48 h of stimulation but only in PC-3 cells (Figure 2D). By contrast, the mRNA levels of *ACACA* were significantly lower under sTWEAK stimulation in the control RWPE-1 after 48 h. No changes were observed in the *ACACA* expression in LNCaP cells. Additionally, no changes were observed for the ATP citrate synthase gene expression (*ACLY*) in any cell model.

Examination of the lipolysis-related genes patatin-like phospholipase domain containing 2 (*PNPLA2*) and carnitine palmitoyltransferase 1A (*CPT1A*) revealed that both were significantly upregulated in PC-3 and LNCaP cells 48 h after sTWEAK administration, but no changes were observed in RWPE-1 cells (Figure 2E).

Other genes involved in lipid metabolism regulation, such as peroxisome proliferator-activated receptor gamma (*PPARG*) and sterol regulatory element binding transcription factor 1 (*SREBP-1*), were exclusively and significantly upregulated by sTWEAK treatment in PC-3 cells at 48 h, whereas *PPARG* was significantly downregulated in RWPE-1 cells (Figure 2F). Regarding lipid transporter-related genes, we observed a significant increase in a cluster of differentiation 36 gene expression (*CD36*) in sTWEAK-treated PC-3 at 48 h, whereas fatty acid-binding protein 4 (*FABP4*) expression was significantly downregulated in LNCaP cells at 24 and 48 h but was upregulated in PC-3 cells at 48 h (Figure 2F). No sTWEAK-induced changes in lipid transporter-related genes were observed in RWPE-1 cells.

A correlation between gene and protein expression under sTWEAK stimulation was verified by Western blotting for the selected lipid metabolism-related genes (Figure S2 and its detailed information can be found in supplementary materials).

### 3.3. Silencing of Fn14 (TNFRSF12A) Abrogates the Ability of sTWEAK to Modulate Lipid Metabolism Genes in PC-3 Cells

**Figure 3:**
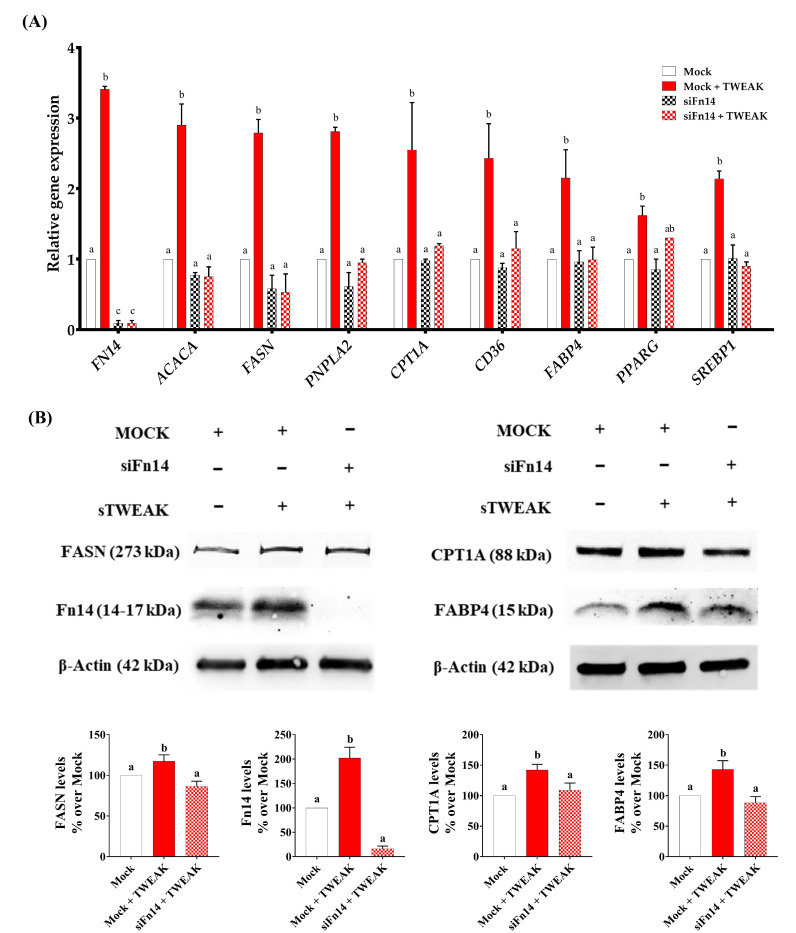
Inhibition of Fn14 in PC-3 cells blocks the sTWEAK-induced expression of lipid metabolism-related genes. (**A**) Fn14 was silenced in PC-3 cells using a Fn14 siRNA; a siRNA-negative sequence (MOCK) was used as a control. The cells were treated with 100 ng/mL TWEAK for 48 h, and the lipid-related gene expression was measured by RT-qPCR. (**B**) Protein expression in Fn14 siRNA/MOCK-transfected PC-3 cells of FASN, CPT1, Fn14 and FABP4 by Western blotting. Detailed information about the Western blotting can be found at Figure S3. Different lettering over the boxes indicates statistical differences. Significant differences are established at *p* < 0.05. Data are expressed as mean ± SEM (*n* = 3 to 4 experiments). Abbreviations: Fn14, fibroblast growth factor-inducible 14; FASN, Fatty acid synthase; CPT1A, Carnitine palmitoyltransferase IA; siFn14, Fn14 small interfering RNA; FABP4, fatty acid-binding protein 4; ACACA, Acetyl-CoA Carboxylase Alpha; PNPLA2, patatin-like phospholipase 2; CD36, cluster of differentiation 36; PPARG, Peroxisome proliferator-activated receptor gamma; SREBP1, Sterol regulatory element-binding transcription factor 1; RT-qPCR, reverse transcription quantitative PCR.

Because lipid metabolism-related genes were significantly upregulated by sTWEAK in PC-3 cells, we chose this line for further analysis. To corroborate that the observed effects on the lipid metabolism were due to TWEAK/Fn14 engagement, we silenced the Fn14 gene (*TNFRSF12A*) using a specific siRNA. The cells were then stimulated with sTWEAK for 48 h. Compared with MOCK-transfected cells, the siRNA-mediated knockdown of the Fn14 gene resulted in the complete reduction of its expression (mRNA and protein) (Figure 3A,B) after sTWEAK stimulation. Additionally, siFn14 treatment also abolished the significant sTWEAK-induced expression of genes involved in lipogenesis (*ACACA* and *FASN*), lipolysis (*PNPLA2* and *CPT1A*), lipid transport (*CD36* and *FABP4*) and lipid regulation (*PPARG* and *SREBP1*) (Figure 3A), clearly indicating that the observed effects of sTWEAK were due to ligand–receptor binding. These effects were paralleled by a reduction in the protein expression of the selected proteins FASN, CPT1A and FABP4, as evaluated by Western blotting (Figure 3B).

### 3.4. sTWEAK Stimulates Fatty Acid Uptake and Lipid Accumulation in PC-3 Cells

**Figure 4:**
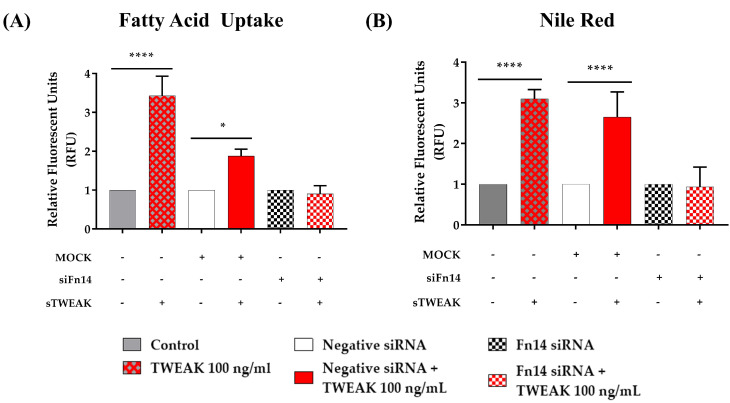
sTWEAK stimulation induces lipid uptake and accumulation in PC-3 cells. Cells were treated with 100 ng/mL TWEAK for 48 h. (**A**) Uptake of the fatty acid analog TF2-C12 was measured after 30 min. (**B**) Lipid accumulation was measured with Nile Red dye after 15 min of incubation. Fn14 was silenced in PC-3 cells using a Fn14 siRNA; a siRNA-negative sequence (MOCK) was used as a control. Data are expressed as mean ± SEM (*n* = 4 experiments). Significant differences: * *p* < 0.05 and **** *p* < 0.0001. Abbreviations: sTWEAK, soluble TNF-like weak inducer of apoptosis; siFn14, Fn14 small interfering RNA.

To directly measure the stimulatory effect of sTWEAK on the lipid metabolism, we utilized lipid uptake assays (Figure 4). As shown in Figure 4A, PC-3 cells treated for 48 h with sTWEAK significantly increased the fatty acid uptake, and this effect was significantly blocked-in cells pretreated with siFn14 before stimulation with sTWEAK. Notably, sTWEAK stimulation also increased lipid accumulation, as shown by Nile Red staining, Figure 4B).

### 3.5. sTWEAK Stimulates Lipid Metabolism Genes in PC-3 Cells by Phosphorylating ERK1/2 and AKT(ser473)

**Figure 5:**
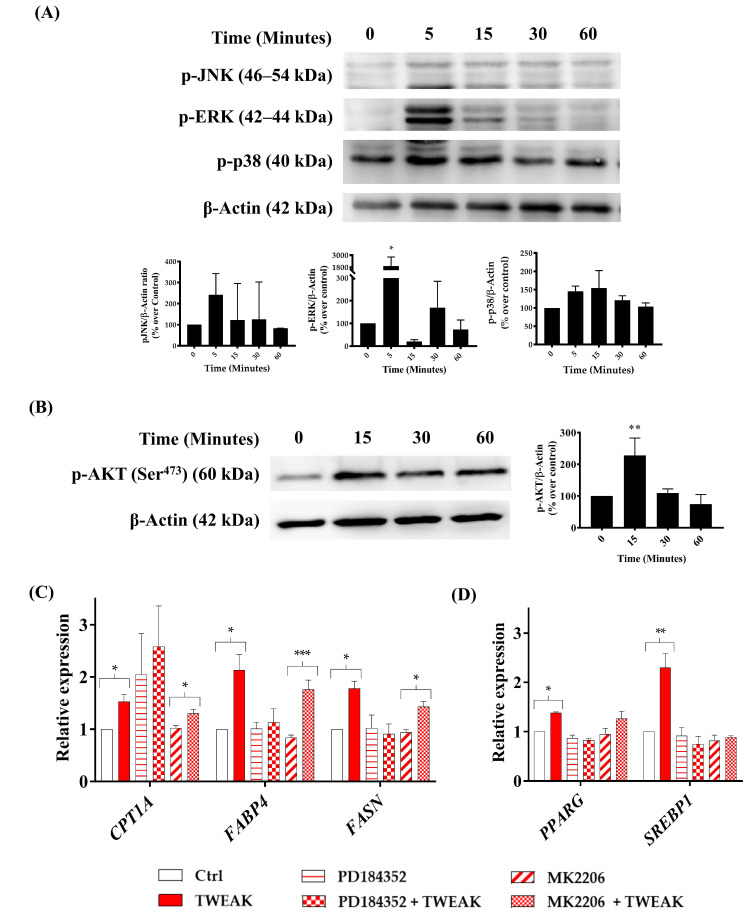
sTWEAK phosphorylates ERK1/2 and AKT(ser473) and regulates lipid-related gene expression in PC-3 cells. Cells were stimulated with 100 ng/mL sTWEAK at different time points. (**A**) MAPK and (**B**) AKT (ser473) phosphorylation were analyzed by Western blotting. Representative Western blots and a densitometry analysis of 3 independent experiments (mean ± SEM) are shown. * *p* < 0.05 and ** *p* < 0.01. PC-3 cells were treated with 10 μM ERK1/2 inhibitor (PD184352) or 1 µM AKT inhibitor (MK2206) and then stimulated with 100 ng/mL sTWEAK for 24 h. The gene expression of the (**C**) *CPT1A*, *FABP4* and *FASN* and (**D**) *PPARG* and *SREBP1* transcription factors were evaluated by RT-qPCR. The relative gene expression levels are shown normalized to their corresponding untreated controls. Data are expressed as the mean ± SEM (*n* = 4 experiments). Significant differences: * *p* < 0.05, ** *p* < 0.01 and *** *p* < 0.001. Detailed information about the Western blotting can be found in Figure S6. Abbreviations: p-JNK, phospho-Jun N-terminal Kinase; p-ERK, phospho-extracellular signal-regulated kinase, p-p38, phospho- p38 mitogen-activated protein kinase, p-AKT, phospho- Protein kinase B; β-Actin, beta-actin; CPT1A, Carnitine palmitoyltransferase IA; FABP4, fatty acid-binding protein 4; FASN, Fatty acid synthase; SREBP1, Sterol regulatory element-binding transcription factor 1; PPARG, Peroxisome proliferator-activated receptor gamma; % over control: values are calculated as percentage over untreated cells at time 0; kDa, Kilodaltons: RT-qPCR, reverse transcription quantitative PCR.

Activation of the phosphatidylinositol 3-kinase (PI3K-AKT-mTORC1) pathway in cancer cells reprograms the cellular metabolism by augmenting the activity of metabolic enzymes [3]. Mitogen-activated phosphokinase (MAPK) signaling pathways have also been implicated in redirecting energy utilization in malignant cells [27]. Examination of MAPK signaling in sTWEAK-stimulated PC-3 cells revealed the activation (phosphorylation) of ERK1/2, but no changes were observed for p38 or JNK1/2 phosphorylation (Figure 5A). We also observed that sTWEAK induced AKT (ser473) phosphorylation in PC-3 cells (Figure 5B). Similar results were observed for LNCaP cells, but AKT (ser473) phosphorylation occurred after a 6-h exposure to sTWEAK (Figure S4 and its detailed information can be found in supplementary materials).

To test whether these activated pathways were implicated in the control of the lipid metabolism genes after sTWEAK/Fn14 engagement, we pharmacologically inhibited ERK1/2 and AKT signaling in PC-3 cells. We observed that the expression of genes implicated in lipolysis (*CPT1*), lipid transport (*FABP4)* and de novo lipogenesis (*FASN*) were abolished only by the ERK1/2 inhibitor (Figure 5C), whereas the expression of the gene transcription regulators *SREBP-1* and *PPARG* were significantly regulated at the transcriptional level by both ERK1/2 (PD184352) and AKT (MK2206) inhibitors (Figure 5D).

sTWEAK/Fn14 binding can also activate NF-ĸB canonical and noncanonical pathways in PC-3 cells (and in LNCaP cells; Figure S4); however, specific inhibitor experiments performed in PC-3 cells showed no implication of these pathways in the lipid metabolism (Figure S5).

## 4. Discussion

PCa remains one of the most prevalent cancers in men, and the accurate staging and detection of recurrent and metastatic disease remains a clinical challenge. Once detected, PCa can be treated with different approaches, including active surveillance, or with more aggressive surgical, radiation-based and other focal therapies [28]. All therapies used may have an impact on health-related quality of life. Most PCa cases follow an indolent course that would not threaten the duration or quality of life of patients, but tumor evolution is difficult to predict, as little is known about the factors that influence the onset of PCa or why some tumors progress from indolent to aggressive disease states.

Evaluating changes in metabolic biomarkers in relation to PCa diagnosis might be important to distinguish their contribution to cancer development. In this sense, we observed that hyperinsulinemia and insulin resistance measured by the HOMA-IR index were associated with PCa, as previously reported by others [29]. Hyperinsulinemia has also been reported as a risk factor in PCa development [8,30]. Additionally, the creatinine levels were higher in patients with PCa, but no association with the risk of developing PCa was observed in our cohort, although this association has been reported [31].

Here, we report that the serum levels of the proinflammatory cytokine TWEAK, which has previously been related to glucose metabolism [18], were lower in patients with PCa than in healthy controls. Interestingly, the reduced levels of sTWEAK together with higher levels of PSA, a higher HOMA-IR index and higher LDL cholesterol levels were associated with PCa in our cohort, indicating that TWEAK might be an important component of this pathology. As far as we know, there is no other evidence to suggest that serum sTWEAK concentrations are linked to PCa.

TWEAK is known to be present in the PCa microenvironment, particularly in semen, where its levels are reduced in high- versus low-risk patients [26]. Additionally, the reduced concentration of sTWEAK was inversely related to the expression of the Fn14 gene, a relationship that could explain an active ligand–receptor activity in situ [26]. The tumor metabolic changes induced by alterations of inflammatory cytokines in PCa have been poorly described. Accordingly, the study of PCa cancer cell metabolism, which is experiencing a renaissance in interest [32], under the effect of sTWEAK, might cast some light on the mechanistic understanding of tumorigenesis. Prostate tumor metabolism has been described to be highly lipogenic and lipolytic [33], with relatively low glucose uptake and glycolysis rates mainly in the early stages of PCa [34] compared with other solid tumors [5].

**Figure 6:**
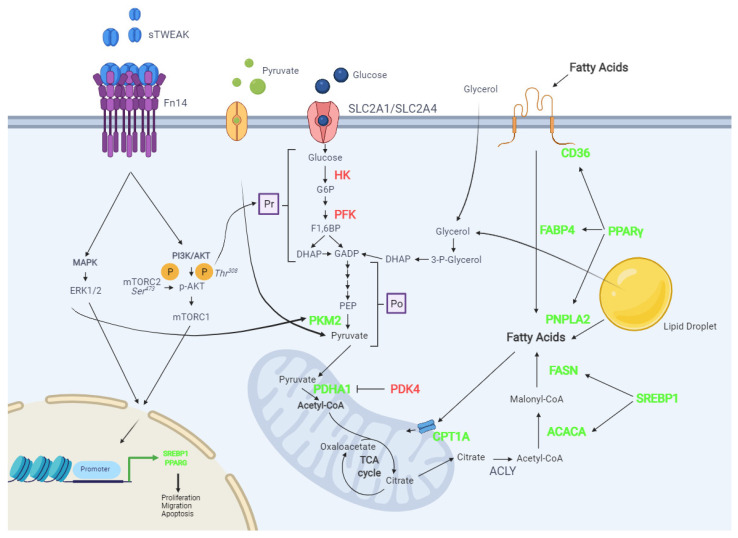
A scheme depicting the possible effects of the TWEAK/Fn14 axis on lipid and glucose metabolic pathways in PCa cells. TWEAK/Fn14 engagement phosphorylates AKT in ser473. PI3K-AKT-mTORC1 signaling is a key factor in controlling lipid and glucose metabolism. pAKT can regulate SREBP1 translocation to the nucleus, increasing ACLY, ACACA and FASN expression, potentiating de novo lipogenesis. AKT phosphorylation can also regulate PPARG, thus increasing the expression of the lipase PNPLA2, which catalyzes the breakdown of the ester bonds of fatty acids and activates lipolysis. Lipid entry and transport is also upregulated by PPARG through *CD36* and *FABP4* expression. CPT1A upregulation by sTWEAK would indicate that lipid oxidation in the mitochondria is boosted. De novo lipogenesis begins with citrate, which is generated by the conversion of pyruvate to acetyl-CoA mediated by an increase in the expression of PDHA1 and a decrease in PDK4 inhibitor expression, both actions regulated by sTWEAK/Fn14 engagement. Phosphorylation of ERK1/2 by sTWEAK/Fn14 engagement can regulate SREBP1 and PPARG and can also increase intracellular pyruvate concentrations by phosphorylating PKM2 and increasing its expression. The preliminary phase of glycolysis might be reduced, since *HK* and *PFK* expression is downregulated. Exogenous pyruvate can also be taken up. Glycerol may be an alternative source for pyruvate. Color pattern: green color represents upregulated genes and red downregulated genes; Created with BioRender.com. Abbreviations: PI3K, Phosphoinositide 3-kinase; AKT, protein kinase B; p-AKT, phosphorylated protein kinase B; MAPK, mitogen-activated protein kinase; ERK, extracellular signal-regulated kinase; TF, transcription factor; SLC2A1, glucose transporter 1, SLC2A4, glucose transporter 4; Pr, preparatory phase; HK, hexokinase; PFK, Phosphofructokinase; G6P, Glucose 6-phosphate; F1,6BP, Fructose-1,6-Biphosphate; DHAP, Dihydroxyacetone phosphate; GADP, glyceraldehyde-3-phosphate; Po, Payoff phase; PEP, phosphoenolpyruvate; PKM2, Pyruvate kinase 2; PDHA1, Pyruvate dehydrogenase; PDK4, Pyruvate dehydrogenase lipoamide kinase isozyme 4; TCA, tricarboxylic acid; ACLY, ATP citrate synthase; ACACA, Acetyl-CoA Carboxylase Alpha; SREBP1, Sterol regulatory element-binding transcription factor 1; FASN, Fatty acid synthase; PNPLA2, patatin-like phospholipase 2; PPARγ, Peroxisome proliferator-activated receptor gamma; FABP4, fatty acid-binding protein 4; CD36, cluster of differentiation 36; CPT1A, Carnitine palmitoyltransferase IA; 3-P-Glycerol, 3-phosphoglycerol.

To test the possible metabolic actions of this cytokine on PCa cells, we used two established in vitro models of PCa disease: PC-3 cells (androgen-independent and invasive) and LNCaP cells (androgen-dependent and noninvasive). As a control for these studies, we used nontumorigenic RWPE-1 prostate epithelial cells [33]. LNCaP and PC3 cell lines can be models for different stages of prostate cancer. Our analysis of glucose metabolism-related genes in the studied cell lines revealed the reduced gene expression in the “preparatory phase” of glycolysis after sTWEAK treatment. By contrast, in PC-3 cells, sTWEAK administration caused a significant upregulation of genes of the “payoff phase”, *PDHA1* and *PKM2*, which may render pyruvate as a substrate and favoring its entry into the mitochondria for conversion into citrate and, ultimately, fatty acids. A possible explanation for the differences in the expression of preparatory- and payoff-phase genes in PC-3 cells might be the use of glycerol for dihydroxyacetone phosphate synthesis and conversion to pyruvate (Figure 6).

Metabolic imaging of PCa tumors with labeled pyruvate [35] has been more useful than glucose, because PCa tumors show a relatively low glucose uptake [36]. Pyruvate can also be obtained by PCa cells through glycerol metabolism, because a higher activity of mitochondrial glycerol-3-phosphatedehydrogenase, which regulates cytosolic glycerol-3-phosphate (G3P), has been detected in PCa [37]. The use of glycerol by PCa cells has been indirectly demonstrated, because the enzyme monoacylglycerol lipase (MAGL), which converts monoacylglycerol into glycerol, has been shown to be upregulated in more aggressive PCa cell lines [38]. Glycerol can also be externally provided to tumor cells by PCa adipose tissue, as adipocytes treated with PCa-conditioned media increase their levels of PNPLA2 and secrete large amounts of free glycerol that may enter the PCa cells, fueling lipid metabolism [39] (Figure 6).

Of note, although sTWEAK does not positively regulate genes implicated in glucose metabolism in the studied PCa cells, glucose uptake experiments have demonstrated a low basal glucose metabolism in PCa cells versus a dominant fatty acid uptake [40,41], suggesting that fatty acids intake should be envisaged as target for further therapeutic PCa treatment [42].

Deregulation of lipogenic enzymes in PCa correlates with worse prognosis and poor survival [43]. sTWEAK treatment of PC-3 cells upregulated de novo lipogenesis, as evidenced by an increase in *ACACA* and *FASN* gene expression. Both genes are involved in the conversion of mitochondrial-derived citrate to fatty acids. This was accompanied by an increase in the expression of master key lipid regulatory genes such as *SREBP-1* and *PPARG* and by upregulation of the lipid membrane transporter gene *CD36* and the cytoplasmic fatty acid-binding protein *FABP4*. Consistent with this finding, we found a concordant sTWEAK-induced increase in cellular lipids and enhanced fatty acid uptake. The upregulation of *FASN* expression by sTWEAK is particularly relevant, as it can act as an oncogene in PCa [44], and FASN protein overexpression has been linked to poor recurrence-free survival in PCa [45]. Fatty acid oxidation is also reported to be deregulated in cancer cells [46], and, in this scenario, sTWEAK might upregulate this lipolytic process by increasing *CPT1* expression in the mitochondria and *PNPLA2* expression in the cytoplasm, both reported to be deregulated in PCa [47,48].

The enhancing effect of sTWEAK on lipid metabolism gene expression in the PC-3 cellular model was mediated by binding to the Fn14 receptor, which was paralleled by an increase in the corresponding protein expression. Overall, our observations suggest that sTWEAK treatment in PC-3 cells stimulates cells to obtain energy by the oxidation of fatty acids. Therefore, since lipid metabolism may confer more aggressive properties to malignant cancers cells [49], sTWEAK may be potentiating an aggressive PCa progression.

Several signaling cascades activated by TWEAK/Fn14 are implicated in PCa, including the canonical and noncanonical NF-ĸB signaling pathways and MAPKs [50]. Of the three known MAPK pathways—extracellular signal-regulated kinases (ERKs), c-Jun N-terminal kinases (JNKs) and p38 kinases [51]—only ERKs and JNKs have been shown to be key metabolic regulators in malignant cells [27]. We found that TWEAK activates the ERK1/2 pathway in PCa cells, influencing the cellular metabolism. This effect could be mediated by phosphorylating PKM2 [27], which, in turn, can regulate SREBP-1 expression, a key regulator of lipid metabolism in several cancer types [52] (Figure 6).

Activation of the PI3K-AKT-mTORC1 pathway is important in the regulation of lipid and glucose metabolisms [53,54]. sTWEAK treatment of PCa cells phosphorylates AKT at serine473, known to be mediated by mTORC2 [55] (Figure 6). The mechanistic links between Akt and SREBP on the lipid metabolism are well-known [56]. Of note, the timing of activation differed between PC-3 and LNCaP, being a late event in the former. This distinctive signaling trait between the two PCa lines in the present study might, in part, explain the differences observed in lipid gene expression.

We are aware that cell line-based studies have inherent limitations, as they likely do not represent the heterogeneity observed in PCa, but they are nevertheless useful tools to explore the mechanisms underlying tumorigenesis and drug resistance. Future studies with patient-derived cancer models, which are more representative of the original tumor [57], will be needed as the next step to explore the mechanistic relationship between the cytokine TWEAK and lipid metabolism in PCa.

## 5. Conclusions

Our results lead us to conclude that reduced serum levels of sTWEAK are independently associated with an increased risk of PCa. Additionally, we established that the presence of sTWEAK in the PCa microenvironment contributes to PCa lipid metabolism by modulating the expression of lipid-related genes, thus potentiating an aggressive progression. Since the inhibition of TWEAK/Fn14 engagement can block this metabolic effect in vitro, targeting Fn14 might be a therapeutic strategy for the management of PCa.

## Figures and Tables

**Table 1:** Anthropometric and analytical characteristics of the studied cohorts.

Number of Patients	Controls	PCa Patients	*p*-Value
	*n* = 159	*n* = 76	
	Mean ± SD	Mean ± SD	
Anthropometric Parameters			
Age (years)	63.54 ± 7.26	63.51 ± 6.66	0.953
BMI (kg/m^2^)	28.60 ± 2.31	28.24 ± 3.97	0.067
**ISUP GG (*n*/%)**	-	-	-
Low Risk (I and II)	-	48 (63%)	-
High Risk (Group III, IV, V)	-	28 (37%)	-
**Glycemic profile**			
Glucose (mmol/L)	5.20 ± 0.53	6.03 ± 1.35	<0.001
Insulin (pmol/L)	30.60 ± 19.93	94.53 ± 55.33	<0.001
HOMA-IR	1.04 ± 0.74	3.75 ± 2.63	<0.001
Diabetes (*n*/%)	0 (0%)	8 (10.6%)	<0.001
**Lipid profile**			
Cholesterol (mmol/L)	5.17 ± 0.99	4.96 ± 1.04	0.233
HDL cholesterol (mmol/L)	1.22 ± 0.28	1.47 ± 0.66	0.001
LDL cholesterol (mmol/L)	2.87 ± 0.71	3.07 ± 1.23	0.185
Triglycerides (mmol/L)	1.50 ± 0.90	1.49 ± 0.81	0.925
**Hepatic profile**			
GGT (µkat/L)	0.64 ± 0.67	0.70 ± 0.79	0.404
**Renal profile**			
Uric acid (µmol/L)	366.46 ± 78.47	413.27 ± 379.57	0.527
Creatinine (μmol/L)	76.74 ± 13.31	70.98 ± 14.21	<0.001
Total PSA (μg/L)	1.64 ± 2.14	10.06 ± 7.73	<0.001
sTWEAK	1583.13 ± 1348.60	1197.20 ± 579.57	<0.001

Abbreviations: PCa, Prostate Cancer; SD, Standard deviation; ISUP (GG): International Society of Urological Pathology Gleason Grading; BMI, Body mass index; cc, centiliters; HOMA-IR, Homeostatic Model Assessment for Insulin Resistance; HDL, High-density lipoprotein; LDL, Low-density lipoprotein; GGT, Gamma Glutamyltransferase; PSA, Prostate-specific antigen; sTWEAK, soluble tumor necrosis factor-like weak inducer of apoptosis.

## Data Availability

The data presented in this study are available on request from the corresponding authors.

## Supplementary Materials

The following are available online at https://www.mdpi.com/article/10.3390/cancers13184688/s1, Figure S1: *FN14* gene expression in all the studied PCa cell lines (PC-3, LNCaP and RWPE-1). Figure S2: sTWEAK on the protein expression of CPT1A, FABP4 and FASN. Figure S3: Complete Western blotting results, referring to Figure 3. Figure S4: Effect of sTWEAK stimulation on several signaling pathways in the PCa cell models. Figure S5: PC-3 cells were treated with inhibitors of noncanonical and canonical NK-ĸB signaling. Figure S6: Complete Western blotting results, referring to Figure 5.
