# Supplementary material for: Serum Levels of the Cytokine TWEAK Are Associated with Metabolic Status in Patients with Prostate Cancer and Modulate Cancer Cell Lipid Metabolism In Vitro
Source: Cancers (Basel). 2021 Sep 18;13(18):4688. doi: 10.3390/cancers13184688 (PMC8465414; doi:10.3390/cancers13184688)
Supplement: Supplementary file 1 [file cancers-13-04688-s001.zip › cancers-1368599-supplementary.pdf]

cancers

Supplementary Materials

Serum Levels of the Cytokine TWEAK Are Associated with Metabolic Status in Patients with Prostate Cancer and Modulate Cancer Cell Lipid Metabolism In Vitro

Antonio Altuna-Coy, Xavier Ruiz-Plazas, Marta Alves-Santiago, José Segarra-Tomás and Matilde R. Chacón

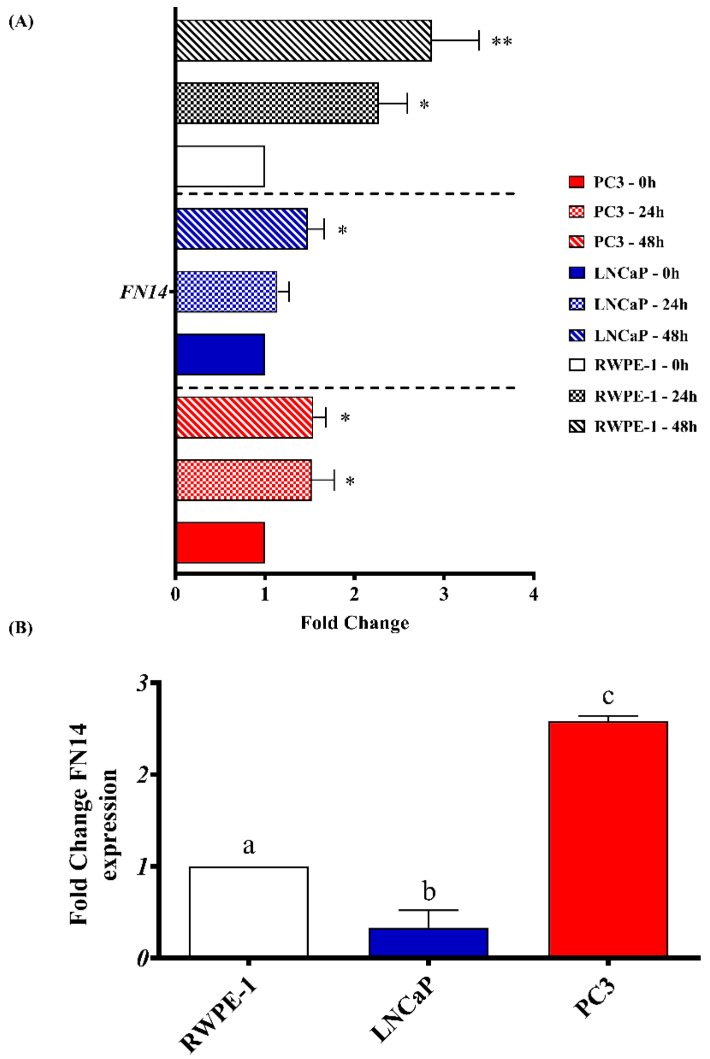

Figure S1. *FN14* gene expression in all the studied PCa cell lines (PC-3, LNCaP and RWPE-1). (**A**) *Fn14* gene expression before and after 100ng/mL sTWEAK treatment at 24 h and 48 h. Fold change represented vs. its corresponding untreated condition. * $p < 0.05$ and ** $p < 0.01$. (**B**) *Fn14* gene expression. Basal levels. Different lettering over the boxes indicates statistical differences. Significant differences are established at $p < 0.05$. Data are expressed as the mean ± SEM ($n = 4$ experiments). Abbreviations: FN14, fibroblast growth factor-inducible 14; PC-3, androgen-independent human advanced adenocarcinoma prostate cancer cell line, LNCaP, androgen-sensitive human prostate adenocarcinoma cell line; RWPE-1 epithelial cell line derived from the peripheral zone of a histologically normal adult human prostate.

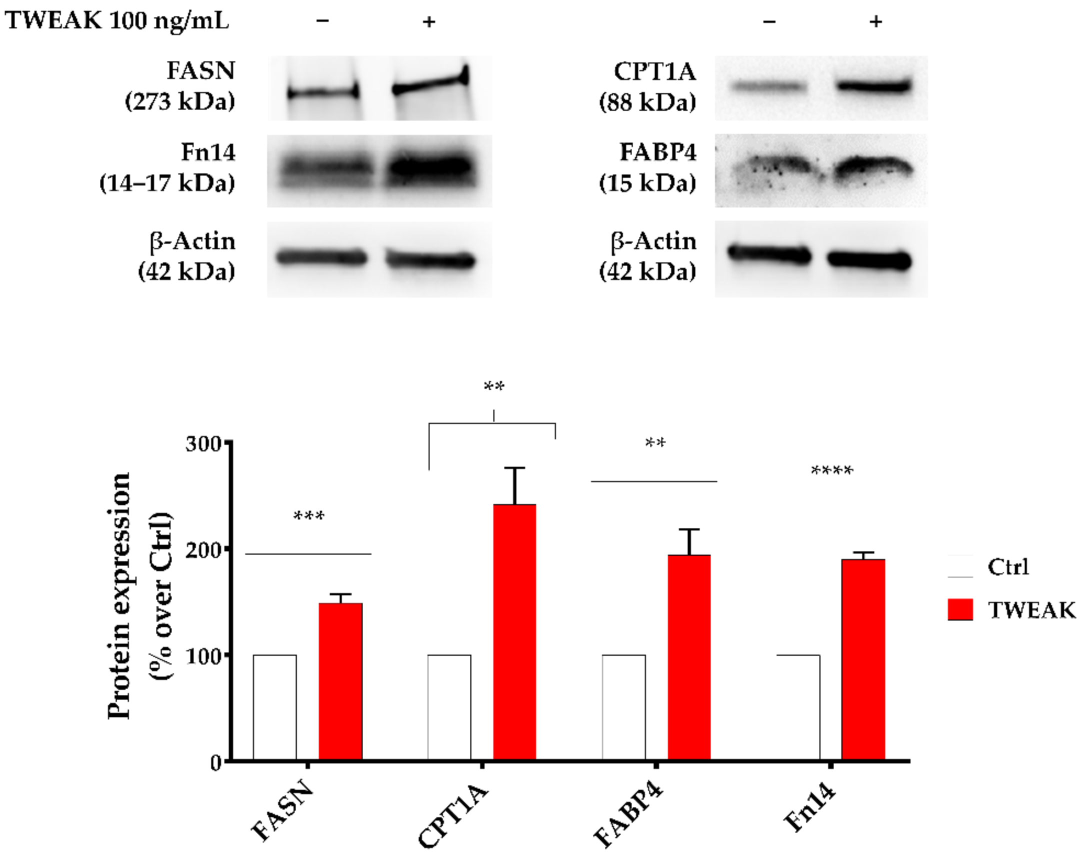

Figure S2. sTWEAK on the protein expression of CPT1A, FABP4 and FASN. PC-3 cells were stimulated with sTWEAK 100 ng/mL during 48 h. The protein was analyzed by Western blotting. ** $p < 0.01$. *** $p < 0.001$, ****$p < 0.0001$. Abbreviations: TWEAK, TNF-like weak inducer of apoptosis; FASN, Fatty acid synthase, CPT1A, Carnitine palmitoyltransferase IA; FABP4, fatty acid-binding protein 4; KDa, Kilodanton; β-Actin, beta-actin; FN14, fibroblast growth factor-inducible 14; % over control: values are calculated as percentage over untreated cells (Ctrl) at time 0.

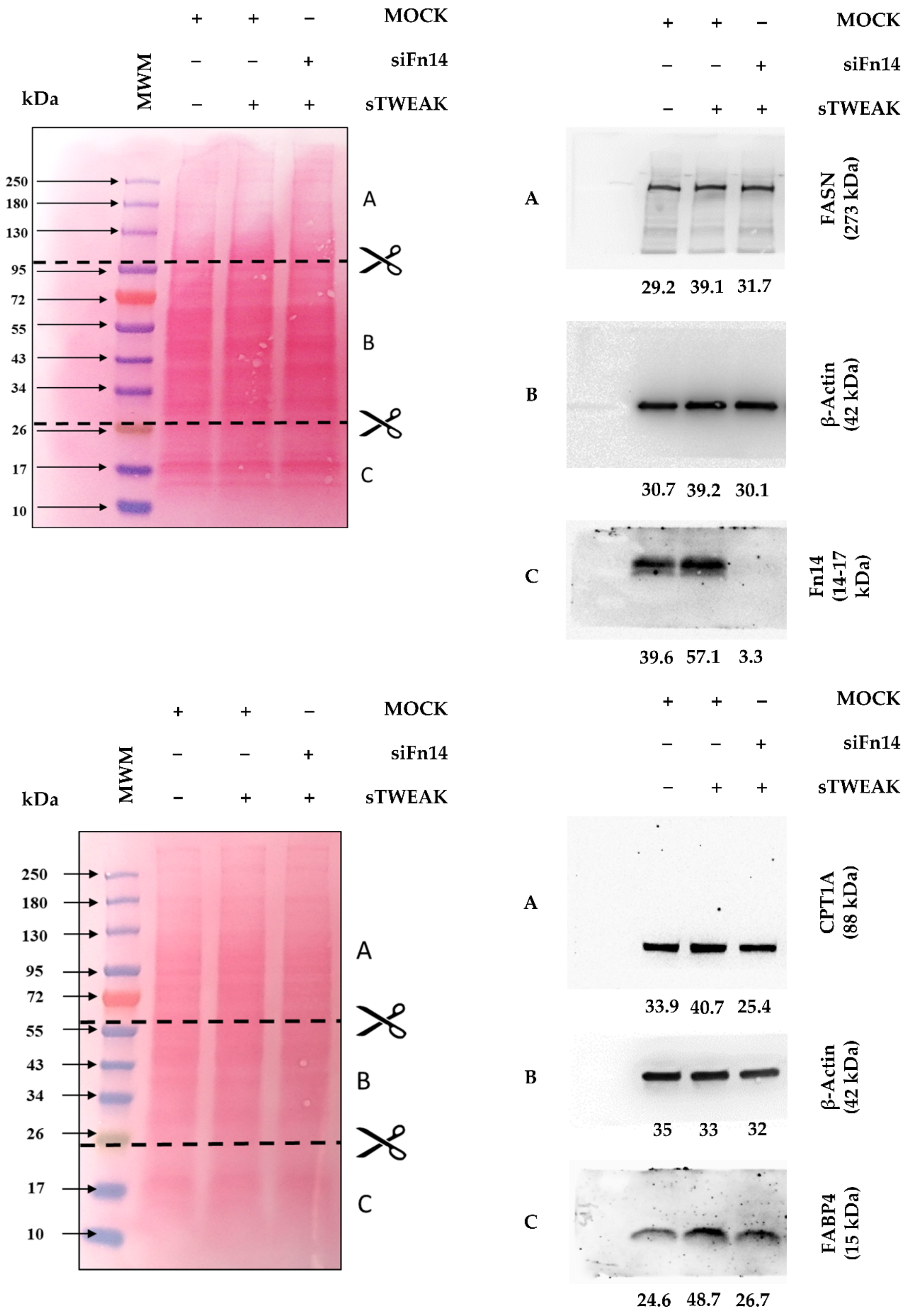

Figure S3. Complete WB results, referring to Figure 3. The numbers below the membranes represent the percentage of the intensity. Abbreviations: MOCK, siRNA negative control; siFN14, Fn14 small interfering RNA, sTWEAK, soluble TNF-like weak inducer of apoptosis; MWM, Molecular Weight Markers; CPT1A, CPT1A, Carnitine palmitoyltransferase IA; Fn14, fibroblast growth factor-inducible 14; FABP4, fatty acid-binding protein 4; FASN, Fatty acid synthase; kDa, Kilodaltons. Symbols: Scissor/dotted lines: position where membrane was cut and incubated with antibodies as indicated in each panel.

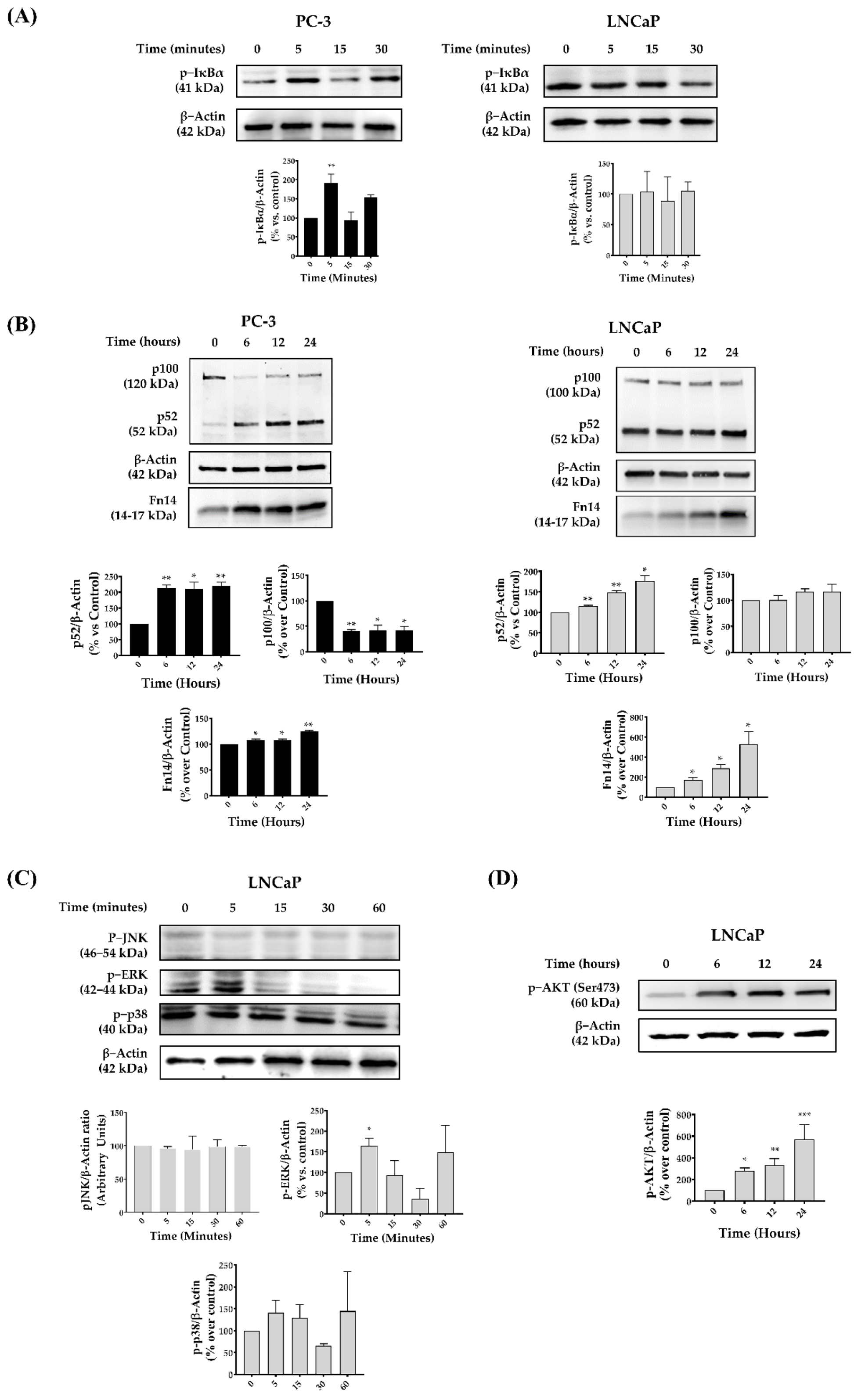

Figure S4. Effect of sTWEAK stimulus on several signaling pathways in the PCa cell models. PC-3 and LNCaP cells lines were stimulated with 100ng/mL sTWEAK at different time points. (**A**) Canonical NF-κB. (**B**) Noncanonical NF-κB. (**C**) MAPK pathway. (**D**) AKT phosphorylation. Representative immunoblots of 4 independent experiments (means ± SEM) are shown. * $p < 0.05$, ** $p < 0.01$, *** $p < 0.001$ and **** $p < 0.0001$. Abbreviations: p-IKBα, phospho-nuclear factor of kappa light polypeptide gene enhancer in B-cells inhibitor, alpha; p-100, Nuclear factor NF-kappa-B p100 subunit; p-52, Nuclear factor NF-kappa-B p52 subunit, β-Actin, beta-actin; Fn14, fibroblast growth factor-inducible 14; p-JNK, phospho-Jun N-terminal Kinase; p-ERK, phospho-extracellular signal-regulated kinase, p-p38, phospho- p38 mitogen-activated protein kinase, p-AKT, phospho- Protein kinase B; % over control: values are calculated as percentage over untreated cells at time 0; PC-3,androgen-independent human advanced adenocarcinoma prostate cancer cell line, LNCaP, androgen-sensitive human prostate adenocarcinoma cell line.

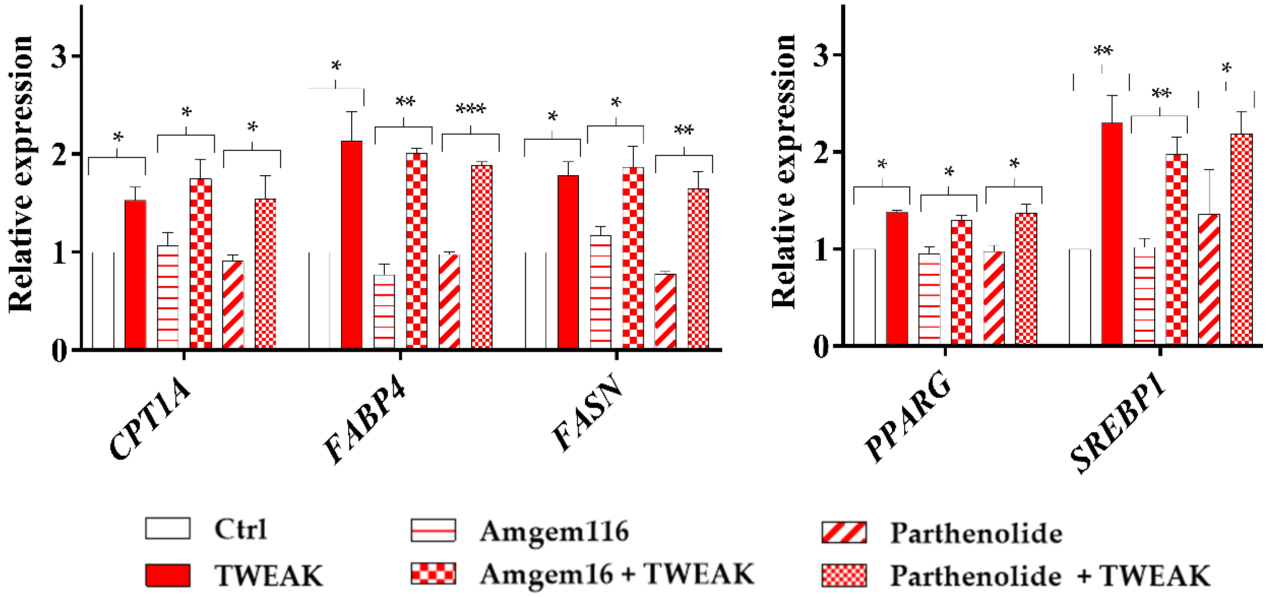

Figure S5. PC-3 cells were treated noncanonical and canonical inhibitors with NF-κB signaling. The cells were treated with 10 μM of a noncanonical NF-κB inhibitor (Amgem) or 10 μM of canonical NF-κB inhibitor (parthenolide) and then stimulated with 100-ng/mL sTWEAK for 24 hours. The gene expression of *CPT1A*, *FABP4*, *FASN* and *PPARG* and *SREBP1* were evaluated by RT-qPCR. The relative gene expression levels are shown normalized to their corresponding untreated control. Data are expressed as the mean ± SEM (n = 4 experiments). Significant differences: * $p < 0.05$, ** $p < 0.01$ and *** $p < 0.001$. Abbreviations: TWEAK, TNF-like weak inducer of apoptosis; CPT1A, Carnitine palmitoyltransferase IA; FABP4, fatty acid-binding protein 4; FASN, Fatty acid synthase; SREBP1, Sterol regulatory element-binding transcription factor 1; PPARG, Peroxisome proliferator-activated receptor gamma; RT-qPCR, reverse transcription quantitative PCR.

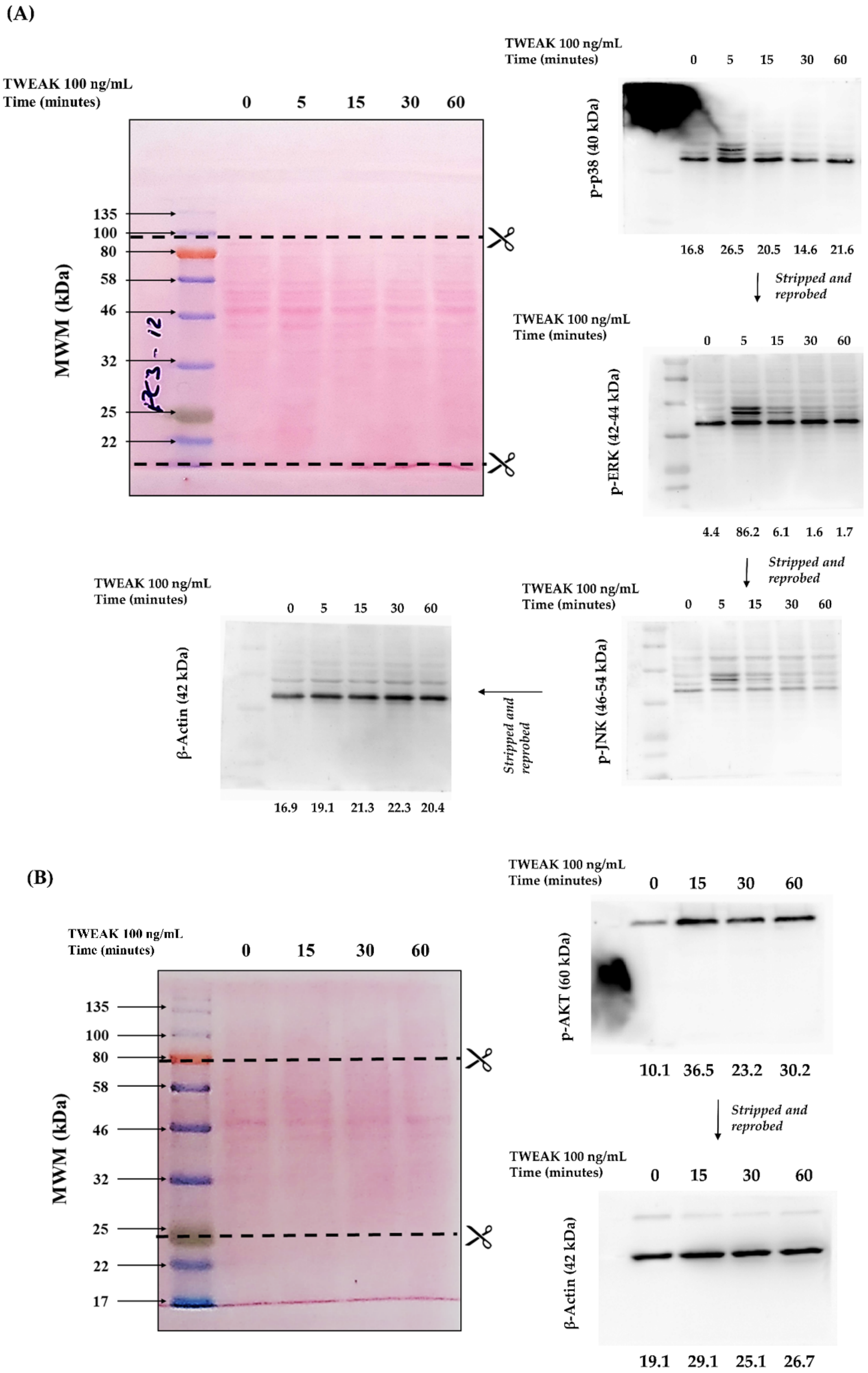

Figure S6. Detail information about Figure 5. The numbers below the membranes represent the percentage of the intensity. Abbreviations: sTWEAK, soluble TNF-like weak inducer of apoptosis; MWM, Molecular Weight Markers; kDa, Kilodaltons; p-JNK, phospho-Jun N-terminal Kinase; p-ERK, phospho-extracellular signal-regulated kinase, p-p38, phospho- p38 mitogen-activated protein kinase, p-AKT, phospho- Protein kinase B; β-Actin, beta-actin Symbols: Scissor/dotted lines: position where membrane was cut and incubated with antibodies as indicated in each panel.

(A)

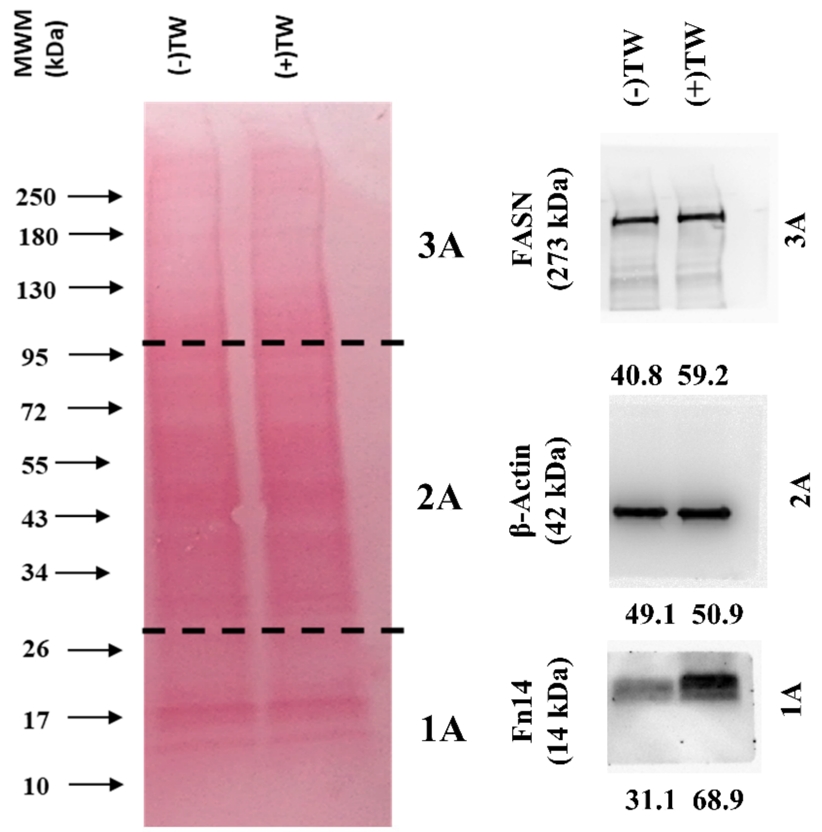

(B)

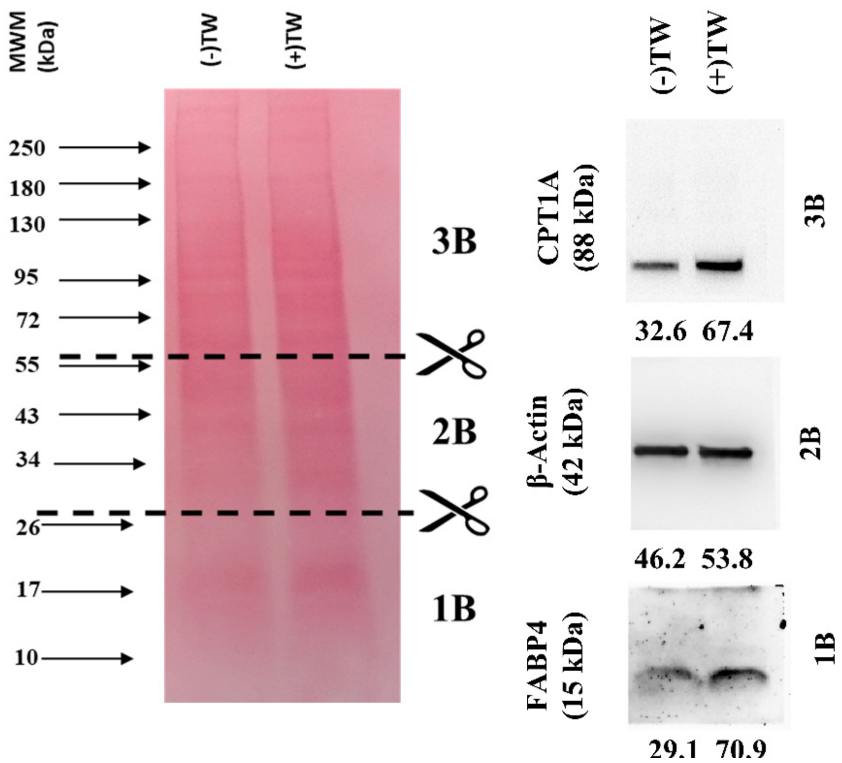

Detailed information about Figure S2 Complete WB results. The numbers below the membranes represent the percentage of the intensity. Abbreviations: (-)TW, untreated with TNF-like weak inducer of apoptosis; (+)TW, treated with TNF-like weak inducer of apoptosis; MWM, Molecular Weight Markers; kDa, Kilodaltons; FASN, Fatty Acid Synthase; Fn14, Fibroblast growth factor-inducible 14; CPT1A, Carnitine palmitoyltransferase I A; FABP4, Fatty acid-binding protein 4. Symbols: Scissor/dotted lines: position where membrane was cut and incubated with antibodies as indicated in each panel.

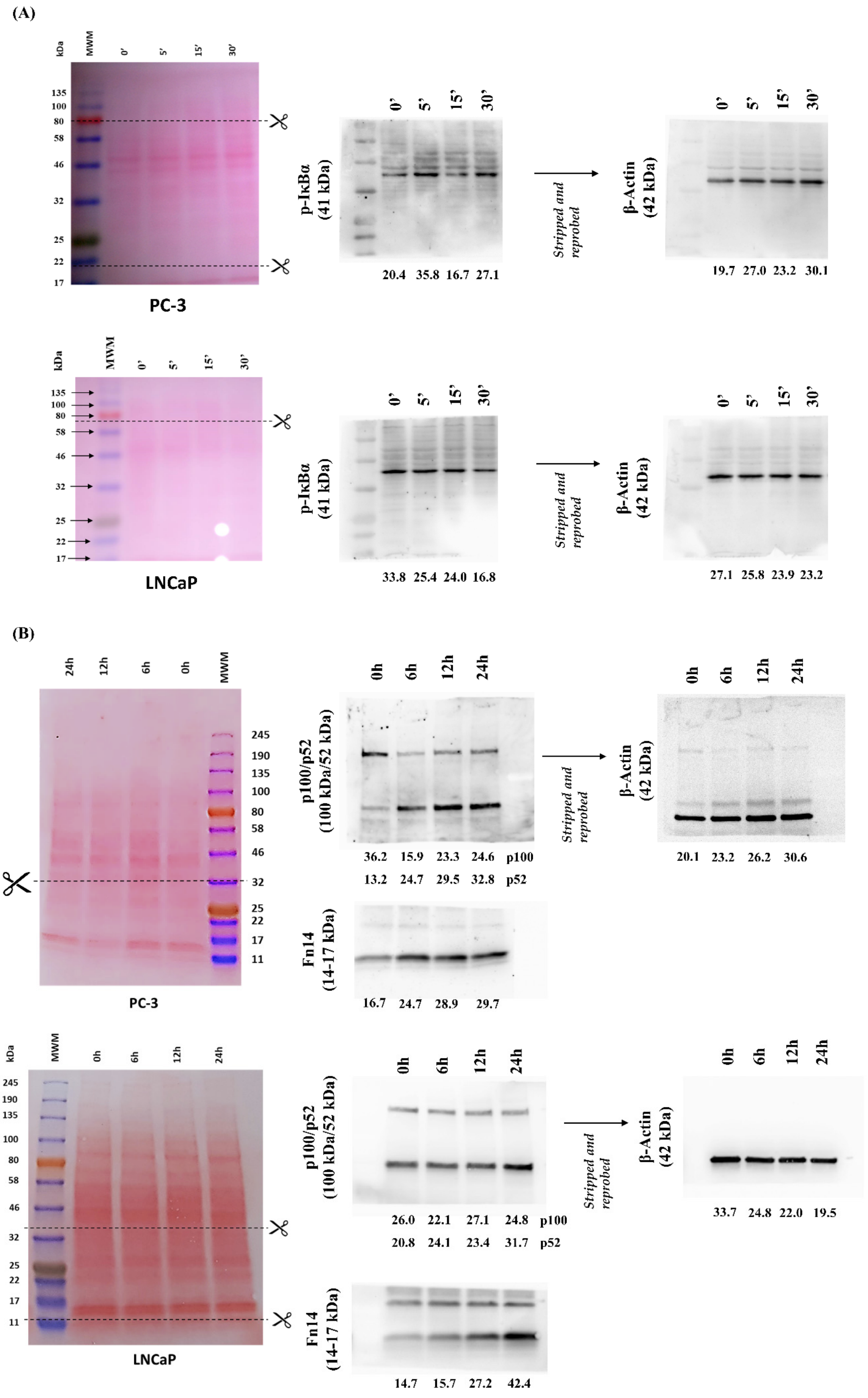
(A)
kDa
MWM
0'
5'
15'
30'
135
100
80
58
46
32
25
22
17
PC-3
p-IκBα
(41 kDa)
20.4 35.8 16.7 27.1
Stripped and reprobed
β-Actin
(42 kDa)
19.7 27.0 23.2 30.1
LNCaP
33.8 25.4 24.0 16.8
27.1 25.8 23.9 23.2
(B)
24h
12h
6h
0h
245
190
11
p100/p52
(100 kDa/52 kDa)
36.2 15.9 23.3 24.6 p100
13.2 24.7 29.5 32.8 p52
20.1 23.2 26.2 30.6
Fn14
(14-17 kDa)
16.7 24.7 28.9 29.7
26.0 22.1 27.1 24.8 p100
20.8 24.1 23.4 31.7 p52
33.7 24.8 22.0 19.5
14.7 15.7 27.2 42.4

(C)

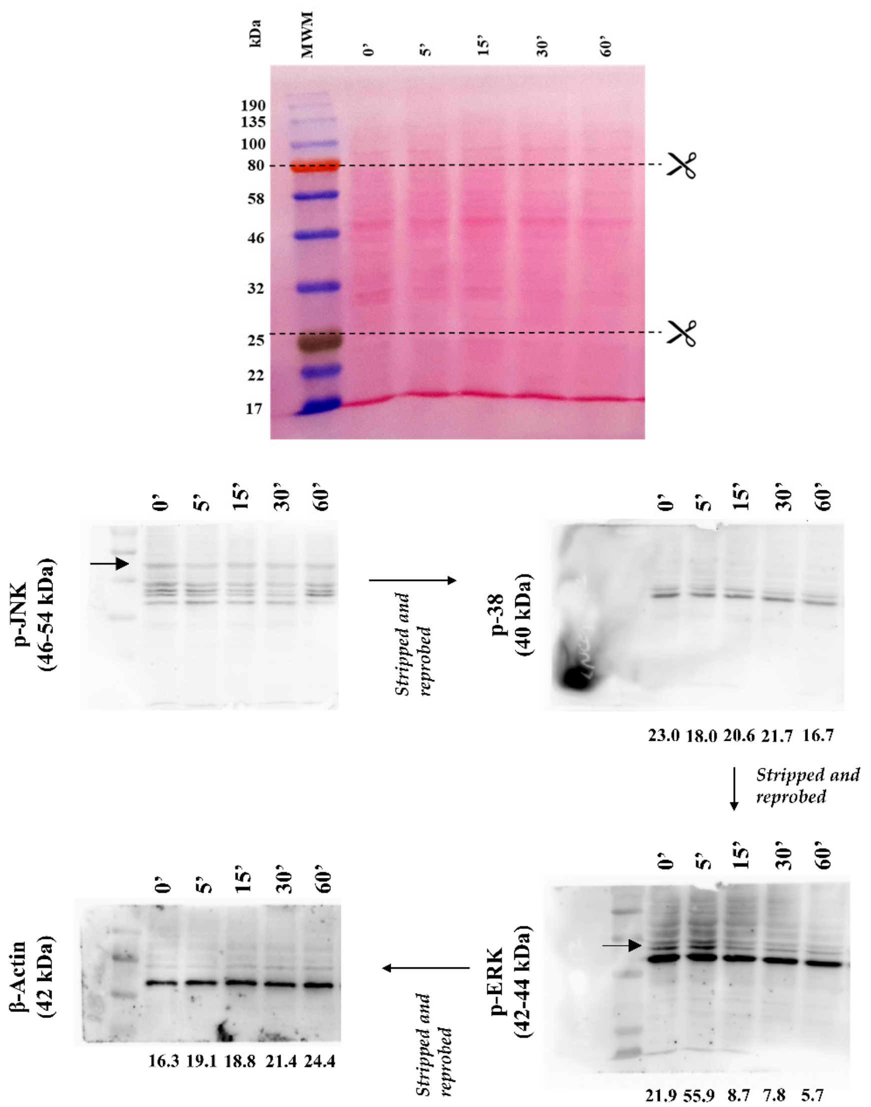

(D)

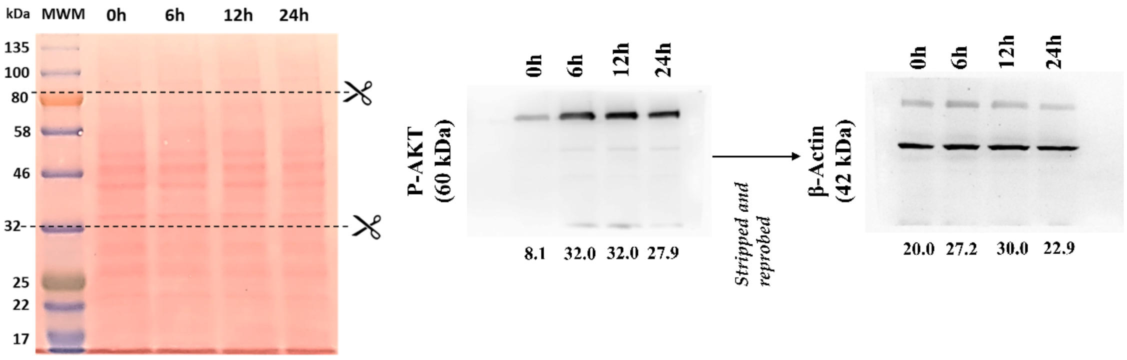

Detailed information about Figure S3. Complete WB results. The numbers below the membranes represent the percentage of the intensity. Abbreviations: MWM, Molecular Weight Markers; kDa, Kilodaltons; p-IKBα, phospho-nuclear factor of kappa light polypeptide gene enhancer in B-cells inhibitor, alpha; p-100, Nuclear factor NF-kappa-B p100 subunit; p-52, Nuclear factor NF-kappa-B p52 subunit, β-Actin, beta-actin; Fn14, fibroblast growth factor-inducible 14; p-JNK, phospho-Jun N-terminal Kinase; p-ERK, phospho-extracellular signal-regulated kinase, p-p38, phospho- p38 mitogen-activated protein kinase, p-AKT, phospho- Protein kinase B; PC-3,androgen-independent human advanced adenocarcinoma prostate cancer cell line, LNCaP, androgen-sensitive human prostate adenocarcinoma cell line. Symbols: Scissor/dotted lines: position where membrane was cut and incubated with antibodies as indicated in each panel.
